# Determination of subtypes of *Blastocystis* sp. in Chilean patients with and without inflammatory bowel syndrome, A preliminary report

**DOI:** 10.1016/j.parepi.2019.e00125

**Published:** 2019-11-21

**Authors:** Sebastián Peña, Gabriela Carrasco, Pamela Rojas, Douglas Castillo, Luiz S. Ozaki, Rubén Mercado

**Affiliations:** a Parasitology Unit, Faculty of Medicine, Universidad de Chile, Santiago, Chile; b Department of Nutrition, Faculty of Medicine, Universidad de Chile, Santiago, Chile; c Parasitology Laboratory, Faculty of Medicine, Universidad de Chile, Santiago, Chile; d Centre for Biological Data Sciences, Life Sciences, Department of Immunology and Microbiology, Virginia Commonwealth University, Richmond, VA, United States of America

**Keywords:** *Blastocystis*, Subtypes, Irritable bowel syndrome, IBS, Chile

## Abstract

**Background & methods:**

*Blastocystis* sp. is one of the most prevalent unicellular eukaryote of the human large intestine in Chile and worldwide. It is classified in subtypes (STs), where using the polymorphic sequences of its *18S rRNA* genes currently recognizes 22. STs 1–9 and ST12 have been reported in humans. It has been hypothesized that different STs of *Blastocystis* sp. differentially affect the clinical severity of the digestive disease in Irritable Bowel Syndrome (IBS) patients, but more studies ar4e needed to establish this statement. To contribute in the elucidation of the potential relationship between *Blastocystis* sp. subtypes and IBS severity, 37 IBS patient fecal samples were collected at hospitals in Santiago (Chile) and were screened for the presence of vacuolated forms of *Blastocystis* sp. by using conventional microscopy. Positive samples were submitted to PCR and sequencing for determining STs. The same procedure was performed in fecal samples from five non-IBS *Blastocystis* sp. carriers for preliminary comparative purpose.

**Results and discussion:**

Four out of the 37 samples from the IBS patients were found positive for *Blastocystis* sp. (10.81%) by using microscopy. The presence of this microorganism in these four samples were confirmed by PCR and sequencing. Subtypes and their respective closest match alleles were searched and the ST1, ST2 and ST4 subtypes were found in these patients. ST4 subtype is scarcely detected in South America countries, being reported previously only in Colombia and Brazil. In this ST4 subtype we determined the allele 42 which is the most frequent allele observed in human *Blastocystis* isolates. In the non-IBS individuals' carriers, three subtypes were found: ST1, ST2 and ST3, even belonging to the same family group. Closest match alleles: 2, 12 and 34 here detected were also commonly reported globally. Instead of the small number of IBS patients studied here, the frequency of blastocystosis detected (10.81%) was lower than the prevalence of *Blastocystis* sp. infections described for the Chilean general population (30.4%). In Chile, clear correlation of *Blastocystis* sp. subtypes and IBS severity is still lacking with this study but it may lead and contribute to a better understanding of its pathogenicity and worldwide epidemiology.

## 1 Introduction

*Blastocystis* sp. inhabits the large intestine of humans and it is the most frequently observed enteric protist in Chile and throughout the world (Mercado and Schenone, 2004; Jiménez et al., 2019). *Blastocystis* sp. is subtyped through sequences of its small subunit ribosomal RNA (*18S SSU rDNA*) gene and classified as STs, being the most common worldwide the ST1, ST2 and ST3 subtypes (Stensvold et al., 2007; Seyer et al., 2016). Subtyping of this enteric protist has been performed in South America, mainly in Argentina, Bolivia, Brazil, Colombia, Ecuador and Peru (Ramirez et al., 2016) but not yet in Chile.

The relationship of *Blastocystis* sp. and Inflammatory Bowel Syndrome (IBS) has been suggested in industrialized countries and in Chile (Ibarra et al., 2016). It has been described that ST1 and ST3 subtypes of *Blastocystis* sp. are frequently detected in IBS patients (Cifre et al., 2018; Rostami et al., 2017). However, the pathogenic role of this organism continues under study (Kurt et al., 2016). Statistics of IBS prevalence in Chile is scarce but a diagnosis of this condition has increased in the last decade. By using the diagnostic criteria and management recommendations of Rome II, IBS was estimated to occur in 28,6% of the adult population of Santiago, the Metropolitan Region of Chile (Ibarra et al., 2016).

In order to investigate the putative involvement of *Blastocystis* sp. subtypes in IBS, we studied 37 IBS patients in Santiago, Chile for this protist, through its *SSU rDNA* subtyping and subtypes alleles. We also determined STs and their respective alleles in fecal samples of five Chilean individuals with blastocystosis that included two children and three adult members of the same family, who had no IBS symptoms.

## 2 Material and methods

### 2.1 Ethics statement

This study was approved under the number 212–2015 by the Ethics Committee on Research in Human Beings of the Faculty of Medicine of the Universidad de Chile.

### 2.2 Samples and patient characterization

Fecal samples of the 37 symptomatic participants of the IBS study group were collected for one year, from September 2017 to September 2018 in Santiago, Chile. All IBS symptomatic patients were adults (average age: 35 years-old) recruited following the Rome IV Diagnostic Criteria for IBS (Drossman and Hasler, 2016; Lacy and Patel, 2017). Five fecal samples of three adults and two children with no IBS symptoms but *Blastocystis* sp. presence in a parasitological microscopically examination was used to determine in non-IBS carriers STs of *Blastocystis* sp. for preliminary comparative purposes. These fecal samples were originally collected in 2018 to detect intestinal parasites in a family group from Santiago and in a parasitological epidemiology survey in children done in La Serena city and donated to us by the respectively researchers.

Fecal samples were divided into two aliquots and transported in plastic containers to the laboratory of the Parasitology Unit in the Faculty of Medicine of the University of Chile for processing. One aliquot was fixed with sodium acetate-acetic acid-formalin (SAF) (Thermofisher Scientific, KS, USA) for microscopy examination and the other preserved in ethanol 70% for molecular diagnostic processing. All samples were stored at 4 °C until use.

### 2.3 Microscopy examination and molecular procedures

SAF fixed samples were examined by light microscopy at 40× (AXIO-Lab.A1, Zeiss, Germany) for *Blastocystis* sp. vacuolar forms (VF) detection. The corresponding positive samples preserved in ethanol were processed for DNA extraction as follows: Feces were concentrated using PARA-PAK (Meridian Inc., USA) and DNA extracted from 250 μl of concentrated feces using the QIAamp DNA Stool Mini Kit (QIAGEN, MD, USA) according to manufacturer protocol. The resulting DNA samples were stored at −20 °C until further processing. PCR was performed in a total volume of 25 μl containing 0.5 μM of each of the primers RD5 5′-ATC TGG TTG ATC CTG CCA GT-3′ and BhRDr 5′-GAG CTT TTT AAC TGC AAC AAC G-3′ (Scicluna et al., 2006), 1× GoTaq® Green Master Mix (Promega Corporation, WI, USA), 1–5 μl of DNA template, using the following program in the Applied Biosystems 2720 Thermal Cycler (Thermofisher Scientific, KS, USA): initial denaturation at 94 °C for 3 min, 35 cycles of, denaturation at 94 °C for 30 s, annealing at 55 °C for 30 s, extension at 72 °C for 30 s and final extension at 72 °C for 5 min. Amplicons were analyzed by agarose 1% gel electrophoresis in 1× TAE (Thermofisher Scientific, KS, USA) and staining with GelRed® Nucleic Acid Gel Stain (Biotium, CA, USA). An amplicon of about 600 bp was generated in all *Blastocystis* sp. positive samples. PCR products were purified (Wizard® SV Gel and PCR Clean-Up System, Promega Corporation, WI, USA) and sent to an external facility for Sanger sequencing. Electropherograms were visualized and edited with MEGA 7.0 (Kumar et al., 2016) and the resulting sequences were identified in GenBank® using BLAST (NHI U.S. National Library of Medicine). *Blastocystis* sp. identified sequences were then compared to sequences in *Blastocystis* sp. Subtype (*18S*) and Sequence Typing (MLST) database (Jolley et al., 2018) for subtyping and identification of respective closest match alleles.

## 3 Results and discussion

*Blastocystis* sp. subtypes ST1, ST2 and ST4 were observed in the samples from IBS patients, while ST1, ST2, ST3 were identified in the non-IBS samples (Table 1). Only an IBS patient presented the ST4 subtype. Interestingly, three of the non-IBS individuals were members of the same family but their samples presented three different *Blastocystis* sp. subtypes, ST1, ST2 and ST3. Representative partial sequences of each subtype of *Blastocystis* sp. Chilean isolates were uploaded to GenBank with accession numbers: MN403319, MN435628, MN435630, MN435631.

**Table 1:** *Blastocystis* sp. subtypes observed in individuals with and without inflammatory bowel syndrome from Chile.

Patient condition	Location (city)	Age (years)	Gender	*Blastocystis* sp.
				Sbtype
IBS	Santiago	34	Female	ST1
IBS	Santiago	28	Female	ST1^1^
IBS	Santiago	27	Female	ST2^2^
IBS	Santiago	44	Female	ST4^3^
Asymptomatic	Santiago	50	Male	ST1
Asymptomatic	Santiago	45	Female	ST2
Asymptomatic	Santiago	25	Female	ST3^4^
Asymptomatic	La Serena	2	Female	ST2
Asymptomatic	La Serena	3	Male	ST3

*Blastocystis* sp. Subtypes and closest match alleles in Chilean human isolates. (Genbank accession numbers: ^1^*MN435630*; ^*2*^*MN435628*; ^*3*^*MN403319*; ^*4*^*MN435631*).

The ST1, ST2 and ST3 subtypes found in Chile were also found in other surveyed South American countries (Ramirez et al., 2016), both in non-IBS individuals and IBS patients while the ST4 subtype was observed in Colombia (Ramirez et al., 2016), Brazil (Valença-Barbosa et al., 2017; Barbosa et al., 2018; Seguí et al., 2018) and now in Chile, in an IBS patient. Subtype 4 was mainly detected in Europe, being identified in 74% of Danish patients infected by *Blastocystis* sp. and with symptoms of acute diarrhea (Stensvold et al., 2011), also, in Spain, this subtype was observed in the 94.1% of symptomatic patients with digestive manifestation surveyed in Valencia (Domínguez-Márquez et al., 2009), and it also was detected in symptomatic humans in different communities of the northern region of this country (Paulos et al., 2018).

All alleles: 2, 12, 34 and 42 we have detected here in respectively ST1, ST2, ST3 and ST4 *Blastocystis* subtypes have been previously described by Ramirez et al. (2016) in several countries of South America and it is the allele often described in Europe for this subtype.

In this study, we found *Blastocystis* sp. in 4 out 37 (10.81%) samples from IBS patients, detecting vacuolar forms of *Blastocystis* sp. by using conventional microscopy. It was previously reported that this diagnostic method have low sensitivity compared with in-vitro culture and molecular diagnostic approach, but it has high specificity for detecting this protist in biological samples (Dogruman-Al et al., 2010), this can be a potential bias of our study trying to determine an epidemiological vision of the prevalence of blastocystosis in symptomatic or asymptomatic individuals. The number of IBS patients found positive for this eukaryote is low compared to the frequency determined in the non-IBS population in Chile (Mercado and Arias, 1991; Mercado et al., 2003). Such low frequency has been previously reported in developing nation environment (Morgan et al., 2012), differently to observations in European countries where *Blastocystis* sp. presence in IBS patients was more frequent than in non-IBS controls (Yakoob et al., 2010). More studies are necessary to clarify this discrepancy. The high global frequency of this eukaryotic microbe in humans is a stimulating reason for additional studies to better define aspects of its diagnosis, pathogenicity and epidemiology.
